# The performance of routine immunization in selected African countries during the first six months of the COVID-19 pandemic

**DOI:** 10.11604/pamj.supp.2020.37.1.26107

**Published:** 2020-09-18

**Authors:** Balcha Girma Masresha, Richard Luce, Messeret Eshetu Shibeshi, Bernard Ntsama, Abubacar N’Diaye, Jethro Chakauya, Alain Poy, Richard Mihigo

**Affiliations:** 1 World Health Organization, Regional Office for Africa, Cite de Djoue, Brazzaville, Congo,; 2 World Health Organization, Inter-Country Support Team for West Africa, Ouagadougou, Burkina Faso,; 3 World Health Organization, Inter-Country Support Team for East and Southern Africa, Harare, Zimbabwe,; 4 World Health Organization, Inter-Country Support Team for Central Africa, Libreville, Gabon

**Keywords:** COVID-19, Africa, immunisation, vaccine, coverage, measles

## Abstract

**Introduction:**

following the declaration of the COVID-19 pandemic on 11 March 2020, countries started implementing strict control measures, health workers were re-deployed and health facilities re-purposed to assist COVID-19 control efforts. These measures, along with the public concerns of getting COVID-19, led to a decline in the utilization of regular health services including immunization.

**Methods:**

we reviewed the administrative routine immunization data from 15 African countries for the period from January 2018 to June 2020 to analyze the trends in the monthly number of children vaccinated with specific antigens, and compare the changes in the first three months of the COVID-19 pandemic.

**Results:**

thirteen of the 15 countries showed a decline in the monthly average number of vaccine doses provided, with 6 countries having more than 10% decline. Nine countries had a lower monthly mean of recipients of first dose measles vaccination in the second quarter of 2020 as compared to the first quarter. Guinea, Nigeria, Ghana, Angola, Gabon, and South Sudan experienced a drop in the monthly number of children vaccinated for DPT3 and/or MCV1 of greater than 2 standard deviations at some point in the second quarter of 2020 as compared to the mean for the months January-June of 2018 and 2019.

**Conclusion:**

countries with lower immunization coverage in the pre-COVID period experienced larger declines in the number of children vaccinated immediately after the COVID-19 pandemic was declared. Prolonged and significant reduction in the number of children vaccinated poses a serious risk for outbreaks such as measles. Countries should monitor coverage trends at national and subnational levels, and undertake catch-up vaccination activities to ensure that children who have missed scheduled vaccines receive them at the earliest possible time.

## Introduction

The World Health Organisation (WHO) African Region adopted a strategic plan on immunization (2014-2020) that aims to improve immunization coverage in all countries in the region; to complete interruption of poliovirus transmission and ensure virus containment; and to attain measles elimination and control other vaccine-preventable diseases [1]. Subsequently, countries have developed national multi-year comprehensive plans for immunization as well as annual program plans which reflect these ambitions and targets. Countries regularly monitor vaccination coverage, disease trends and other programmatic indicators at national and subnational levels in order to determine progress towards the set milestones and targets. During major natural disasters, humanitarian crises, armed conflicts and large-scale disease epidemics, national health systems struggle to maintain the provision of routine health services. Community access and utilization of health services also declines in these conditions. The experiences of the various countries have demonstrated the adverse effects of conflicts and large-scale epidemics on health service delivery and access [2-5]. During the 2014-2015 Ebola outbreak in West Africa, it was estimated that antenatal care coverage decreased by 22%, while there was an 8% decline in health facility delivery services, and 13% reduction in postnatal care services [5,6]. The Ebola outbreak also significantly affected measles vaccination coverage rates particularly in Guinea and Liberia where decreases in routine immunization coverage led to high measles incidence that persisted for two years after the end of the Ebola outbreak. Liberia and Guinea experienced a 30% and 33% respective decline in the number of children receiving measles doses in 2014 and a decline of 25% and 26% respectively during 2015 [7]. In addition to measles, Guinea also experienced an outbreak of circulating vaccine derived polio virus (cVDPV) type-2 following the program gaps created during the Ebola outbreak [8].

On March 11, 2020 the COVID-19 outbreak was declared a pandemic by WHO with a total of 118,000 cases reported in 114 countries globally. At the time, nine countries in the WHO African region had reported a total of 47 confirmed COVID-19 cases, with no deaths [9]. By 31 March 2020, 42 countries in the region had reported 3766 COVID-19 cases and 95 COVID-19 deaths [10]. By 30 June 2020, all 47 countries in the region have been affected with a cumulative total of 303,986 COVID-19 cases and 6,155 deaths [11]. As the COVID-19 pandemic continued and increasingly strict control measures including lock-downs and social distancing were imposed in various countries, disruptions of regular preventive and curative health services were reported. Fear of contracting COVID-19 in health facilities, reassignment of health workers, closure of health facilities and stock-out of supplies contributed to this disruption. Routine childhood immunization is among the essential health services that faced disruptions. In addition, previously scheduled mass vaccination campaigns against measles, yellow fever and polio were postponed in a number of countries [12-14]. Statistical models developed taking into consideration the COVID-19 pandemic have attempted to quantify the number of additional deaths expected as a result of the reduced coverage of various child and maternal health interventions. One model projects as much as a 45% increase in under-five childhood deaths and a 39% increase in maternal death per month if coverage of basic life-saving interventions are extensively disrupted [15].

Considering the risk of increased deaths from preventable causes in the context of COVID-19, the World Health Organisation (WHO) developed guidance for countries to ensure the continuity of essential health interventions including immunization, including the appropriate measures to prevent COVID-19 transmission in health care settings [16-18]. Countries monitor the administrative vaccination coverage for each antigen by documenting the number of doses of vaccine delivered in each service delivery site during a defined period, usually one month. These figures are then progressively aggregated at the district, provincial and national levels. The national EPI program shares the compiled country data with the WHO as a monthly report detailing the monthly number of children vaccinated by antigen and by district [19]. This paper examines the actual routine immunization program performance in selected countries in the African Region by comparing the number of children vaccinated in the early months of the COVID-19 pandemic to the number vaccinated in the months prior to the arrival of COVID-19 in the countries.

## Methods

We selected 15 countries in the African Region, seven of which are countries with sustained high immunization coverage in previous years, while the rest are countries with low program coverage as evidenced by the WHO-UNICEF annual vaccination coverage estimates (WUENIC) for 2019 and the immunization program maturity grading conducted for countries in the region [20,21]. In addition, the selection considered countries of different sizes and from different geographic sub-regions of the continent. We reviewed the administrative reporting data from the routine immunization programs for the period from January 2018 to June 2020 to analyze the trends in the monthly number of children vaccinated with specific antigens. We compared the number (monthly average and statistical deviation) of children who received vaccine doses before and after the onset of the COVID-19 pandemic. For this analysis, we reviewed the number of children who received BCG vaccine, the first and third doses of Diphtheria-Pertussis-Tetanus containing vaccine (DPT1 and DPT3) and the first and second doses of measles containing vaccine (MCV1 and MCV2). We also calculated the incidence of COVID-19 at the end of June 2020, using official country reports to the WHO as of 30 June 2020, against the UNPD official population estimates for each country.

## Results

The 2019 WHO/UNICEF coverage estimates used for the selection of the countries are indicated in Table 1, indicating one subset of countries with low coverage, and the remaining countries having high coverage. The number of COVID-19 cases reported in the respective countries is indicated in Table 2, along with the intensity of COVID-19 transmission as of as of the end of June 2020. With the exception of Eritrea and Rwanda having sporadic cases, the other countries had either community transmission or clusters of cases. The highest incidence among this group of countries was in Gabon, followed by the Central African Republic (CAR), Ghana, Guinea and Senegal (Table 2). Most countries in the region detected their first cases of COVID-19 in March 2020.

**Table 1:** WHO-UNICEF coverage estimates for DPT 3 and MCV1 for 2019 in countries selected for the study

Performance category	Country	WHO-UNICEF coverage estimates 2019	
		MCV1	DPT 3
Low coverage	Angola	51%	57%
	CAR	49%	47%
	Chad	41%	50%
	DR Congo	57%	57%
	Gabon	62%	70%
	Guinea	47%	47%
	Nigeria	54%	57%
	South Sudan	49%	49%
High coverage	Burundi	92%	93%
	Eritrea	99%	95%
	Ghana	92%	97%
	Kenya	89%	92%
	Rwanda	96%	98%
	Senegal	90%	93%
	Tanzania	88%	89%

**Table 2:** COVID-19 incidence and transmission status in selected countries as of end June 2020

Country	Reported COVID cases as of end June 2020	COVID transmission status as of end June 2020	UNPD estimated total population (2020)	COVID incidence per million population (as of end June 2020)
Angola	276	Cluster of cases	32,866,268	8.4
CAR	3,613	Community transmission	4,829,764	748.1
Chad	866	Community transmission	16,425,859	52.7
DR Congo	6,938	Community transmission	89,561,404	77.5
Gabon	5,394	Community transmission	2,225,728	2423.5
Guinea	5,351	Community transmission	13,132,792	407.5
Nigeria	25,133	Community transmission	206,139,587	121.9
South Sudan	2,006	Cluster of cases	11,193,729	179.2
Burundi	170	Cluster of cases	11,890,781	14.3
Eritrea	191	Sporadic cases	3,546,427	53.9
Ghana	17,351	Community transmission	31,072,945	558.4
Kenya	6,190	Community transmission	53,771,300	115.1
Rwanda	1,001	Sporadic cases	12,952,209	77.3
Senegal	6,698	Community transmission	16,743,930	400.0
Tanzania	509	Community transmission	59,734,213	8.5

**Figure 1:**
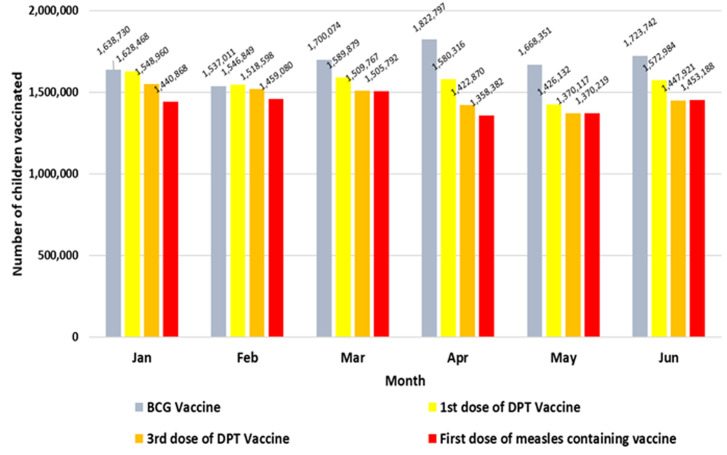
aggregate number of children vaccinated with specific antigens in the selected countries in the African Region, January - June 2020

The completeness of district reporting of immunization data for the first 6 months of the years 2018 - 2020 was >95% in all countries except for South Sudan, which had a completeness of 93% and 96% in 2018 and 2019, while completeness for the first half of 2020 was 91%. The aggregate number of children vaccinated by month in these 15 countries is shown in Figure 1. In April and May of 2020, the number of children vaccinated with DPT1, DPT3 and MCV1 declined as compared to the first quarter of the year. The lowest number of children vaccinated with DPT 1 and DPT 3 doses were in the month of April, while May had the lowest number for MCV1 doses. The number of children vaccinated with DPT3 and MCV1 showed an increase in June as compared to April and May.

At country level, the difference in the mean number of children vaccinated monthly with DPT3 in the months April - June 2020 as compared to January - March 2020 showed a range from a decline of 52% in Guinea to an increase of 6% in Chad. Thirteen of the 15 countries showed a decline in the monthly average of vaccinated children, with 6 countries (Angola, Gabon, Guinea, Nigeria, Burundi and Senegal) having more than 10% decline (Table 3). For the first dose of measles vaccine (MCV1), nine of the 15 countries had a lower monthly mean for April - June as compared to the first quarter of 2020, with Gabon, Guinea, Nigeria and Burundi having a decline in excess of 10%. The change in the mean number of children receiving MCV1 during the second quarter ranged from a decline of 53% in Guinea to an increase of 13% in Chad as compared to the first quarter of 2020 (Table 3). Angola, South Sudan, Eritrea and Kenya, experienced decreases in the number of children vaccinated with DPT3, but increases in the number vaccinated with MCV1. Guinea, Nigeria, Ghana, Angola, Gabon, and South Sudan experienced a drop in the monthly number of children vaccinated for DPT3 and/ or MCV1 greater than 2 standard deviations at some point in the second quarter of 2020 as compared to the mean for the months January - June of 2018 and 2019.

**Table 3:** change in the mean number of monthly DPT 3 and MCV1 doses provided in the first two quarters of 2020

Country	Monthly mean number vaccinated with DPT3		Monthly mean number vaccinated with MCV1			
	January - March 2020	April - June 2020	Percentage change in the two quarters	January - March 2020	April - June 2020	Percentage change in the two quarters
Angola	59058	51686	-12%	62303	66218	6%
CAR	11054	10698	-3%	11418	11074	-3%
Chad	47760	50862	6%	40534	45836	13%
DR Congo	288971	292601	1%	290961	297689	2%
Gabon	3280	2362	-28%	3693	2203	-40%
Guinea	35352	16850	-52%	35605	16788	-53%
Nigeria	560428	492483	-12%	523869	455412	-13%
South Sudan	20885	19404	-7%	15633	16965	9%
Burundi	31923	28210	-12%	35642	28678	-20%
Eritrea	7291	6658	-9%	6766	6912	2%
Ghana	95358	91967	-4%	93035	89433	-4%
Kenya	104171	102379	-2%	100209	110483	10%
Rwanda	28193	27642	-2%	31243	30037	-4%
Senegal	51663	44539	-14%	41719	39486	-5%
Tanzania	180385	175292	-3%	175928	176713	0%

For the entire January - June 2020 period, Burundi, CAR, Chad, DR Congo, Eritrea, Rwanda, and Senegal have maintained the cumulative number of vaccinated children for BCG, DPT1, DPT3 and MCV1, as compared to the mean for the same period in 2018 and 2019. On the other hand, Kenya and South Sudan had measles vaccination figures lower than two standard deviations of the mean in January and February of 2020, while Tanzania had a similar decline in January 2020 (Table 4 and Table 5). Regarding the second dose of measles vaccine (MCV2) provided in the second year of life, Eritrea and Ghana reported a decline in the monthly number of children vaccinated, with the number in April 2020 exceeding 2 standard deviations as compared to the mean for the months January - June of 2018 and 2019 (Table 6). Rwanda, Nigeria and Angola were excluded from this analysis for different reasons though they have MCV2 in their immunization schedules. Rwanda introduced MCV2 starting in 2015, but the country started reporting MCV2 as part of the monthly data shared with WHO only in 2020. Nigeria started providing MCV2 in late 2019, while the monthly data for Angola had wide discrepancies that rendered the trends in 2020 difficult to interpret as compared to the mean for previous years.

**Table 4:** comparison of the monthly number of DPT 3 vaccinated children in January - June 2020 against the monthly mean for the first half of 2018 and 2019

Country	Mean (SD) monthly vaccinated for first half of 2018 - 2019	Monthly number vaccinated with DPT 3 in 2020					
		Jan	Feb	Mar	Apr	May	Jun
Angola	62896 (SD 6003)	66,619	54,727	55,827	44,197	49,351	61,509
CAR	9685 (SD 1545)	10,946	11,175	11,042	11,310	11,118	9,665
Chad	46317 (SD 7538)	46,480	46,238	50,562	50,684	49,611	52,291
DRC	284581 (SD 10005)	287,417	287,931	291,566	295,712	289,302	292,790
Gabon	3667 (SD 337)	3,560	3,456	2,825	1,776	2,203	3,106
Guinea	34997 (SD 1749)	34855	36241	34960	30117	15365	5068
Nigeria	576082 (SD 44967)	577617	565658	538011	493784	459075	524591
S Sudan	18780 (SD 3445)	13752	23212	25692	24808	25940	7464
Burundi	29434 (SD 3191)	34,066	29,115	32,588	32,110	26,230	26,291
Eritrea	6772 (SD 591)	6767	7257	7849	6238	6684	7053
Ghana	94768 (SD 2369)	97066	95303	93707	86779	91075	98048
Kenya	106712 (SD 6479)	107203	98456	106856	99368	99318	108453
Rwanda	28304 (SD 923)	29312	27426	27841	27686	27219	28023
Senegal	48097 (SD= 6198)	54154	51993	48842	44808	42205	46606
Tanzania	168330 (SD 10170)	179146	180410	181599	173493	175421	176963

**Table 5:** comparison of the monthly number of MCV 1 vaccinated children in January - June 2020 against the monthly mean for the first half of 2018 and 2019

Country	Mean number (and standard deviation) of MCV1 recipients for first half of 2018 - 2019	Monthly number vaccinated with MCV1 in 2020					
		Jan	Feb	Mar	Apr	May	Jun
Angola	67199 (SD 11791)	68,203	61,534	57,171	44,988	67,247	86,419
CAR	9357 (SD 1428)	10,886	11,270	12,097	11,910	11,314	9,998
Chad	39897 (SD 5229)	36,220	38,987	46,394	44,626	44,464	48,418
DRC	278615 (SD 13337)	286,911	293,031	292,940	300,137	294,501	298,428
Gabon	3623 (SD 274)	3,944	3,984	3,151	1,867	1,936	2,805
Guinea	35152 (SD 2079)	35311	36498	35008	30155	15119	5091
Nigeria	529868 (SD 47767)	543628	537219	490760	448365	426119	491752
S Sudan	22587 (SD 1833)	9171	17803	19986	21406	22034	7457
Burundi	30298 (SD 3157)	38,699	31,081	37,147	32,967	25,937	27,131
Eritrea	6853 (SD 601)	6037	7069	7194	5983	7180	7575
Ghana	93565 (SD 3248)	95849	92871	90386	80631	93352	94317
Kenya	107599 (SD 6437)	78287	71029	151312	106485	110783	114181
Rwanda	29548 (SD 1784)	34021	29813	29896	31339	29971	28802
Senegal	41326 (SD 5729)	44845	43092	37220	31637	36382	50440
Tanzania	170044 (SD 8674)	148856	183799	195130	165886	183880	180374

**Table 6:** comparison of the monthly number of MCV2 vaccinated children during January - June 2020 against the monthly mean for the first half of 2018 and 2019

Country	Mean number (and standard deviation) of MCV2 recipients for first half of 2018 - 2019	Monthly number vaccinated with MCV2 in 2020					
		Jan	Feb	Mar	Apr	May	Jun
Burundi	23985 (SD 2794)	28,663	21,453	28,644	28,778	22,335	24,532
Eritrea	5738 (SD 379)	5462	6204	5746	4943	6205	6386
Ghana	82154 (SD 3474)	82111	80211	75536	72869	84997	82422
Kenya	64833 (SD 7264)	38519	36690	80085	60532	68087	73562
Senegal	36653 (SD 5983)	43437	40748	34905	30728	29896	35746
Tanzania	139899 (SD 13592)	113942	142462	149603	134659	148263	147012

## Discussion

This analysis has found that the reduction in the number of children vaccinated through the routine immunization programs in 15 countries during the early period of the COVID-19 pandemic varies considerably. Countries with previously high immunisation coverage, such as Senegal, Rwanda and Eritrea have managed to maintain the levels of service delivery according to the reported cumulative number of children vaccinated for the January-June 2020 period. On the other hand, countries with lower program coverage like Gabon, Guinea, Angola and South Sudan experienced larger declines in the number of children vaccinated immediately after the COVID-19 pandemic was declared. Where the reduction in the number of children vaccinated has persisted, as in Guinea, the risk for outbreaks such measles, is expected to persist and increase with time. The absence of large declines in the number of children vaccinated in countries with chronically low coverage such as CAR, Chad, DR Congo may be explained by the differing extent to which countries have been affected by COVID-19 or by the absence of lengthy or strict societal lockdown and movement restrictions. By June 2020, the number of children vaccinated per month does not appear to have recovered to pre-pandemic levels in CAR, Guinea and South Sudan, while for Nigeria, Angola and Ghana only a transient reduction in the number of children was observed.

Countries with weaker health systems are particularly vulnerable to health service disruptions caused by outbreaks, natural disasters, protracted armed conflict or civil disturbances [4]. During the ebola outbreak of 2014 - 2015, Sierra Leone, Liberia and Guinea experienced significant decline in health service delivery [6]. The reduction in measles vaccination documented during the Ebola outbreak in West Africa are comparable to the declines with the COVID-19 outbreak in Guinea. In the two years following the vaccination coverage declines associated with the Ebola outbreak, Liberia, Guinea and Sierra Leone experienced various measles outbreaks that led to a sustained increase in measles incidence despite mass vaccination campaigns and outbreak response immunization efforts [7]. Furthermore, in addition to the disruptions in routine immunization service delivery, many countries have postponed previously scheduled follow-up measles immunization campaigns. These COVID-19 induced delays will further exacerbate the accumulation of susceptible young children, and risks causing measles outbreaks [13]. WHO guidelines recommend a risk-benefit analysis be conducted and the implementation of mass vaccination activities with appropriate infection control measures when the risk of outbreaks is high and further postponement is deemed likely to contribute to outbreaks and deaths from vaccine preventable diseases [18].

The desire to decrease COVID-19 propagation in health facilities as well as the repurposing of health workers may have led to hesitation to continue the usual health facility-based routine immunization services in the early days of the COVID-19 pandemic. However, a benefit-risk analysis has shown that the deaths prevented by sustaining routine childhood immunisation in Africa far outweigh the excess risk of COVID-19 deaths associated with vaccination clinic visits, especially for the vaccinated children [22]. Countries experiencing declines in the coverage of essential health services in the first half of 2020 should ensure the continuity of essential services by implementing the necessary COVID-19 prevention measures and assuring the public about the safety of service delivery. WHO has published guidance on the continuity of essential health services, conducting routine immunization services as well as the considerations when implementing mass vaccination campaigns during the COVID-19 pandemic [17,18]. These guidelines emphasize the need to maintain the provision of immunization services with appropriate precautions to prevent the spread of COVID-19 and a case-by-case assessment of the national COVID-19 and VPD situation. In addition, in August 2020, WHO developed a draft guideline for catch-up vaccination in order to ensure eligible individuals who miss routine vaccine doses for any reason can be identified and vaccinated at the earliest opportunity [23].

In the face of the COVID-19 pandemic, Ethiopia conducted a national measles campaign in July 2020, while DR Congo conducted measles outbreak response and mop-up vaccination in the months of March - June 2020. Both countries undertook these exercises, taking into account the local COVID-19 transmission, and implementing specific COVID-19 prevention measures. There is no evidence to date that these mass vaccination activities contributed to further spread of COVID-19 in the respective countries [24].

Our study has limitations. The analysis considers only 3 months (April-June) of routine immunization administrative coverage data from 15 of 47 countries in the African Region, after the initial detection of confirmed COVID-19 cases in March 2020. Therefore, the trends represented over these three months are only an indication of the early changes in the patterns of immunization coverage. In some countries, administrative coverage data may also include data from periodic intensive service delivery efforts organized in the form of mini-campaigns in order to close service gaps. As a result, the data may reflect the performance beyond the day-to-day service delivery. In addition, only a limited sample of all antigens in routine immunization programs was considered for our analyses. DTP1, DTP3, MCV1 and MCV2 were considered since these antigens are often considered to measure immunization program performance.

## Conclusion

The impact of the COVID-19 pandemic on the provision of routine childhood vaccination services in the first three months following the pandemic has varied by country according to the administrative data available. A universal decline was not observed in all countries studied. Countries should monitor trends in the number of children vaccinated at national and subnational levels, and those that experience extensive and persistent declines should undertake catch-up vaccination activities to ensure that children who have missed scheduled vaccine doses receive them at the earliest possible time. Mass vaccination campaigns may be undertaken in the context of COVID-19, in order to prevent the accumulation of non-vaccinated children and thus avert outbreaks, but only after ensuring that benefit-risk analysis is done, and with the appropriate infection prevention measures. Since countries with low immunization coverage have experienced greater COVID-19 related declines in the number of vaccinated children, they should update their outbreak risk assessments, maintain active disease surveillance and prepare to take measures to mitigate the risk for outbreaks.

### What is known about this topic

- Natural disasters, civil conflict and large epidemics affect health service delivery and utilization;
- The Ebola outbreak of West Africa resulted in serious declines in routine immunisation coverage and subsequent outbreaks of measles and polio in the affected countries;
- Modelling data has shown that the COVID-19 pandemic may affect health service delivery significantly and result in increased child and maternal deaths in low and middle income countries.

### What this study adds

- Countries with strong immunisation programs have not experienced significant reduction in the number of vaccinated children in the three months following the COVID-19 pandemic;
- Countries with lower program coverage experienced larger declines in the number of children vaccinated immediately after the COVID-19 pandemic was declared;
- The observed decline in routine immunization coverage was mainly in the months of April and May 2020, with slight increase observed in June.
